# Effects of the obstruction of erector spinae plane in affected people undergoing percutaneous vertebroplasty

**DOI:** 10.1186/s12893-023-02055-x

**Published:** 2023-06-03

**Authors:** Peng Ju, Dianming Jiang

**Affiliations:** Department of Orthopedics, The Third Affiliated Hospital of Chongqing Medical University, Shuanghu Branch Road, Huixing Street, Yubei District, Chongqing, 401120 China

**Keywords:** Osteoporotic vertebral compression fracture, Percutaneous vertebroplasty, Erector spinae plane block

## Abstract

**Background:**

We aimed to compare the difference between the therapeutic effects of percutaneous vertebroplasty (PVP) as well as PVP combined with erector spinae plane blocked (ESPB) in osteoporotic vertebral compression fractures (OVCFs) therapy.

**Methods:**

After the reception, 100 affected people to OVCFs were randomly divided into the PVP group as a control as well as the PVP + ESPB group as the observation, which included fifty affected people per group. The visual analog scale (VAS) for pain as well as the Oswestry Disability Index (ODI) per group was assessed before the operation, two hours after the operation, and when patients were discharged from the hospital. Operating time was also evaluated on the charged bulk of bone cement during the surgery, blood loss during the surgery, as well as operating costs for each group. Additionally, to assess differences, comparisons have been done among available groups in terms of ambulation as well as defecation or stool after the operation at the earlier time.

**Results:**

The PVP + ESPB category acquired lower VAS and ODI scores when assessments were processed 2 h after the operation and when they were discharged from a hospital. They also had earlier postoperative ambulation and defecation time than the category of PVP (p < 0.05). Regarding the other indicators, there did not show significant differences. Besides, no complications occurred within both group, either after the operation or when they discharge from the hospital.

**Conclusion:**

PVP + ESPB for OVCF is related to less VAS, further effective alleviation of pain, and fewer ODI values in affected people after the operation than only PVP. Besides, affected people can involve in ambulation more swiftly. The PVP + ESPB therapy improves the quicker recuperation of intestinal function as well as helps to improve the overall life quality of patients.

## Background

Because of the aging society, the incidence range of osteoporotic vertebral compression fractures (OVCFs) has grown significantly in the past few years. Due to its subtle resumption rate and shortage of consultation, this condition severely threatens elder people’s health, and it has also further prevalence in women who are in the postmenopausal period [1–3].

Given the existing clinical issues, further research into OVCF has become essential and required [4, 5] The majority of people who are affected by osteoporotic vertebral compression fractures exhibit lower pain in their back as well as worsened postural alterations with spinal deformity. In severe cases, there is decreased cardiopulmonary function, gastrointestinal instability, and even spinae nerve compression, which might result in paralysis as well as some conditions such as life-threatening [6] These days for people who suffered from OVCFs, spinae practitioners more often than not rely on percutaneous vertebroplasty (PVP) to relieve pain. However, this procedure often remains in muscular aches and back pains which are far from the vertebrae that are injured [7] To this end, devising novel approaches for enhancing pain relief in patients is an issue that requires attention. Erector spinae plane block (ESPB) engages the injection of an anesthetic medicine in the shape of local profound to the spinae erectors surface as well as transversal process clearance (at the root superior to the transverse process) along with the surveillance of ultrasonography or G-arm radiography system deployment. The drugs diffuse in the space of the paravertebral as well as block the posterior spinae nerve branches, and probably the anterior spinae nerve branches and relative branches [8] To alleviate the distal engagement pain of the injured spine as well as improve the primary practical workout of affected people, it has been decided to perform ESPB in patients who had undergone PVP. The ESPB process of the majority obstructs the posterior branch of the spinae nerve that fundamentally innervates the responsible fragment of the erector spinae muscle. This subsequently provides alleviation for residual muscular aches and pains after the PVP procedure, alleviating postoperative pain and thereby promoting improvement more swiftly [9, 10] The authors accomplished anatomical localization of 2 formalin-soaked bodies at Chongqing 3 Gorges Medical College in April 2021 to perusal the posterior spinae nerve branch course innervating the erector spinae muscle. Additionally, we recruited 100 affected people by OVCF for this study in six months from July to December 2021 at Chongqing University three Gorges Hospital to investigate the effects of ESPB.

## Methods

### Anatomical utilization

**Fig. 1:**
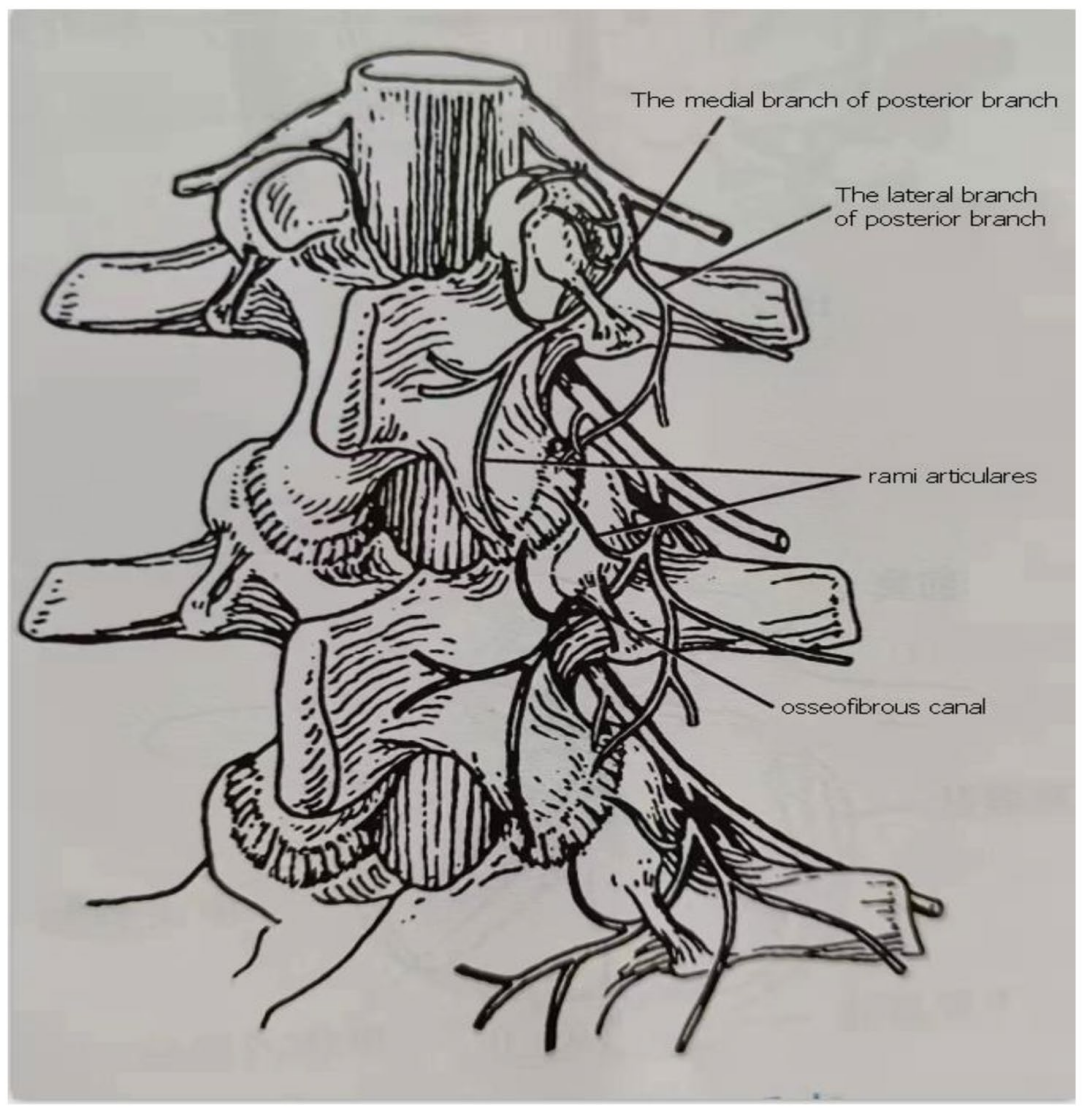
Schematic diagram

The location of the erector spinae muscle which is also recognized as sacrospinalis is in the length of the posterior of the torso as well as in the groove on each side of the spine. This begins from the posterior side of the sacrum, and the back sector of the iliac crest expands superior to the three categories of muscle bundles, also terminates at the vertebrae as well as ribs, across the innervating by the posterior branch of the spinae nerve [11] The posterior branch of the spinae nerve is mixed and tenuous and moves normally in the posterior and lateral position of the upward articular process to the adjacent transverse process, since then dropping into medial and lateral branches except for the sacral nerve [12] Positioned in the epicondyles, at the surfaces of articular, and intervertebral ligaments of the spine, the medial branch innervates the erector spinae, multifidus, and piriformis of the spinae muscles. To a large extent, innervating accomplish by the lateral branch for both the longest muscle of the erector spinae as well as the iliopsoas muscle (Fig. 1) [13].

**Fig. 2:**
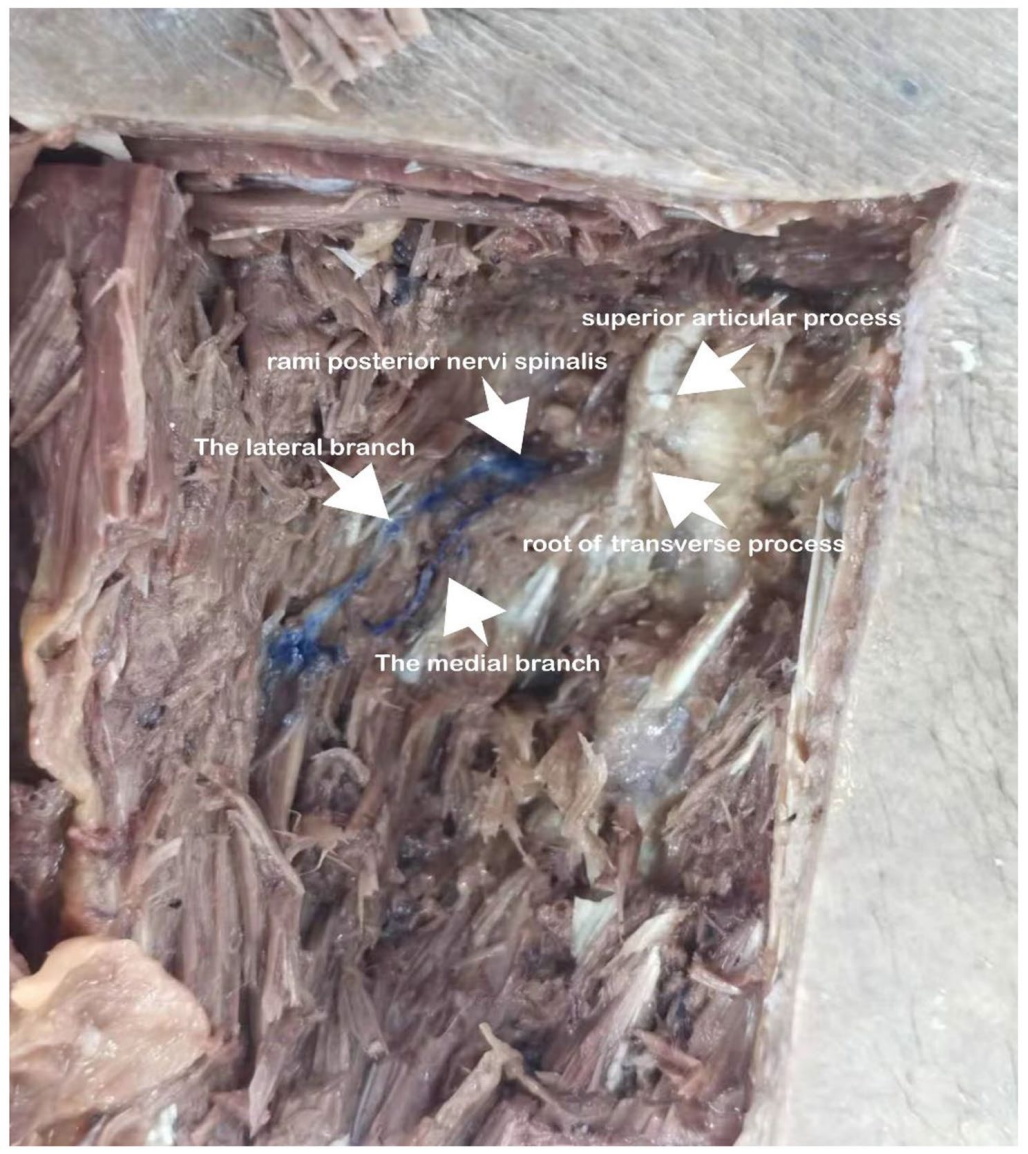
The posterior branch of the spinae nerve, which emanates along the root above the transverse process then falls into medial and lateral branches

As Fig. 2 shows, we performed an autopsy in April 2021 to determine the location of the posterior spinae nerve branch course at Chongqing Three Gorges Medical College.

### Cases and the strategies of experiment

From July to December 2021, we recruited 100 affected people with osteoporotic vertebral compression fractures at Chongqing University Three Gorges Hospital. The patients’ ages were between 60 and 90, with 83 affected individuals who were female as well as 17 affected males. The hospital ethics committee reviewed their cases (Chinese Clinical Trial Registry: ChiCTR2200056202;01/02/2022). Entry criteria: (1) ages equal or above 60; (2) having no records of severe trauma, MRI suggests fresh spine fractures (T2 image of high signal and T1 image of low signal), and without occupying and compressing of the canal of spinae; (3) bone density examination (dual-energy photon evaluation), recommending bone density less or equal to -2.5 SD; (4) Injured vertebrae in one or two parts, and the range of injured vertebrae is T5-L5. Elimination criteria: (1) spinae tumor pathological fractures; (2) patients are unconscious or are not capable to have effective communication; (3) Greater than two injured vertebrae segments. Eligible cases admitted to the hospital were distributed to two categories randomly by lottery, observation category, and control category, and a consent form has been signed by all affected people.

### Study methods

PVP + ESPB observation category: First, the procedure of PVP was carried out on affected people who were positioned in the prone. The injured vertebra using G-arm radiography apparatus positioning, therefore, permitted the designation of the entry points of the bilateral needle. Furthermore, sterilization and draping are routinely conducted. Anaesthesia, in the form of local, was administered with 3ml of lidocaine (2%) at the point of puncture, as well as a cm incision which was made at that point with a sharp blade or knife. The puncture was located in the incision and its location was then adjusted. The G-arm radiography was applied to affirm the location of the head of a needle, and it was placed at the edge of the vertebral frame on the posterior side and the flank position, at the internal edging of the root shadow of the arch. The puncture needle cannula cleared and the working cannula was positioned alongside the other needle as a guide. The G-arm X-ray was used to ensure reaching the tip of the drill on the anterior middle 1/3 of a vertebral body. Removing the drill allowed the mixing of the bone cement ((polymethyl methacrylate). As long as the bone cement become the consistency of toothpaste, a proper amount (the G-arm radiography system displayed injured vertebra was filled with bone cement) was deposited via the aid of the working cannula.

Next, therapy for ESPB was carried out in the injured vertebrae, involving the administration of 5 ml of ropivacaine (Naropin, which was produced in ten ml: 75 mg as well as 0.75% concentration by AstraZeneca AB) for blocking the posterior branch of the spinae nerve bilaterally at the root superior the transverse function of injured vertebral. This procedure has been done by the G-arm aid, and local disinfection with iodine has been done for the incision, returning the patient to the ward after the operation has been done following the covering incision with a dressing.

The category of PVP control:

PVP control group: common PVP operation therapy was accomplished for the applicable injured vertebrae.

### Data and relevant scores

The original data were collected and scored by nurses in the Chongqing University Three Gorges Hospital, while the nurses were blind. For each group, they evaluated the scores of the visual analog scale (VAS) for pain and the Oswestry Disability Index (ODI) before surgery, two hours following surgery, and when they were discharged from the hospital. We also recorded the surgery time spent on repletion of the volume of bone cement during the surgery, blood loss during the surgery, operation costs, and the complication which stem from surgery for each category, as well as the early ambulation time after the operation and the time of defecation (movement of the bowel), as well.

### Statistical analysis

The figures were analyzed by utilizing statistical software, SPSS 25.0, and also the measurement of the data was accomplished by T-test among these groups. Moreover, a p-value less than 0.05 signified a statistically significant difference.

## Results

100 affected people by OVCFs were joined for this experiment, with the observation category containing 50 PVP + ESPB (observation group) as well as the control category including PVP (CONTROL GROUP). The operation has been done successfully for all affected people in both groups, without any death or rehabilitation. Besides, complication after surgery has not been seen in each group. Analysis was performed by SPSS 25.0 statistical software, without statistical differences between these groups regarding VAS and ODI scores before the surgery. However, these two groups were shown some differences in terms of statistics in the scores of VAS and ODI, when observed 120 min after the operation, and also while they exist from the hospital. Statistically significant differences existed between these two categories in early-time ambulation and defecation after surgery.

Statistic differences between these two categories were negligible from the point of the duration time of surgery, repletion of volume of bone cement during the surgery, blood loss during the surgery, as well as operation costs. The particular outcome is indicated in the below tables (Tables 1, 2, 3 and 4).

**Table 1:** VAS scores of each group at different periods

Indexes	Subgroups (mean ± standard deviation)		T	P
	Control (n = 50)	Observation (n = 50)		
Preoperative VAS (score)	7.88 ± 0.69	7.96 ± 0.70	-0.576	0.566
VAS 2 h Preoperative (score)	2.78 ± 0.74	1.32 ± 0.51	11.504	0.000*
VAS at hospital discharge (score)	2.40 ± 0.76	1.06 ± 0.24	11.947	0.000*

*p < 0.05 illustrates a statistically remarkable difference

**Table 2:** ODI scores of each group at different periods

Indexes	Subgroups (mean ± standard deviation)		T	P
	Control (n = 50)	Observation (n = 50)		
Preoperative ODI (%)	76.84 ± 10.74	78.71 ± 9.13	-0.936	0.352
ODI (%) 120 min following the operation	39.91 ± 7.99	17.61 ± 5.43	16.314	0.000*
ODI (%) at hospital discharge	23.01 ± 5.89	11.17 ± 4.93	10.909	0.000*

* p < 0.05 illustrates a statistically remarkable difference

**Table 3:** After surgery ambulation time as well as defecation (stool) time per category

Indexes	Subgroups (mean ± standard deviation)		T	P
	Control (n = 50)	Observation (n = 50)		
ambulation time after surgery(h)	5.17 ± 0.88	2.64 ± 0.48	17.831	0.000*
defecation time (stool) after surgery (h)	15.83 ± 1.33	10.38 ± 1.64	18.246	0.000*

* p < 0.05 illustrates a statistically remarkable difference

**Table 4:** Comparison of intraoperative conditions in each group

Indexes	Subgroups (mean ± standard deviation)		T	P
	Control (n = 50)	Observation (n = 50)		
operation time (min)	44.46 ± 13.77	47.72 ± 15.27	-1.121	0.265
bone cement volume (ml) during surgery	4.55 ± 1.55	4.65 ± 1.19	-0.361	0.719
blood loss (ml) during surgery	2.40 ± 1.28	2.56 ± 1.20	-0.646	0.520
Operation expense (Yuan)	2110.17 ± 253.62	2082.84 ± 239.76	0.554	0.581

*p < 0.05 illustrates a statistically remarkable difference

**Fig. 3:**
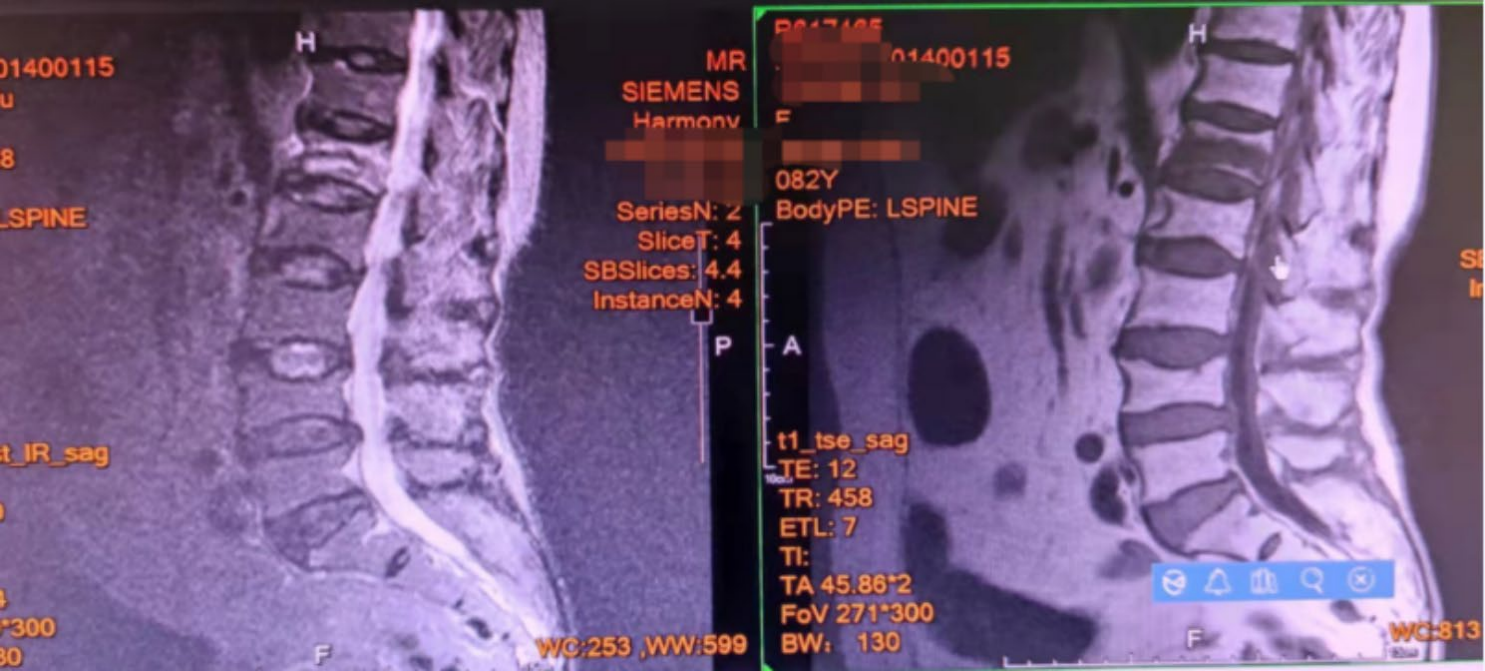
MRI of the lumbar spine suggests a high signal in the T2 image, a low signal in the T1 image, and a fresh fracture of the lumbar 1 vertebral body

**Fig. 4:**
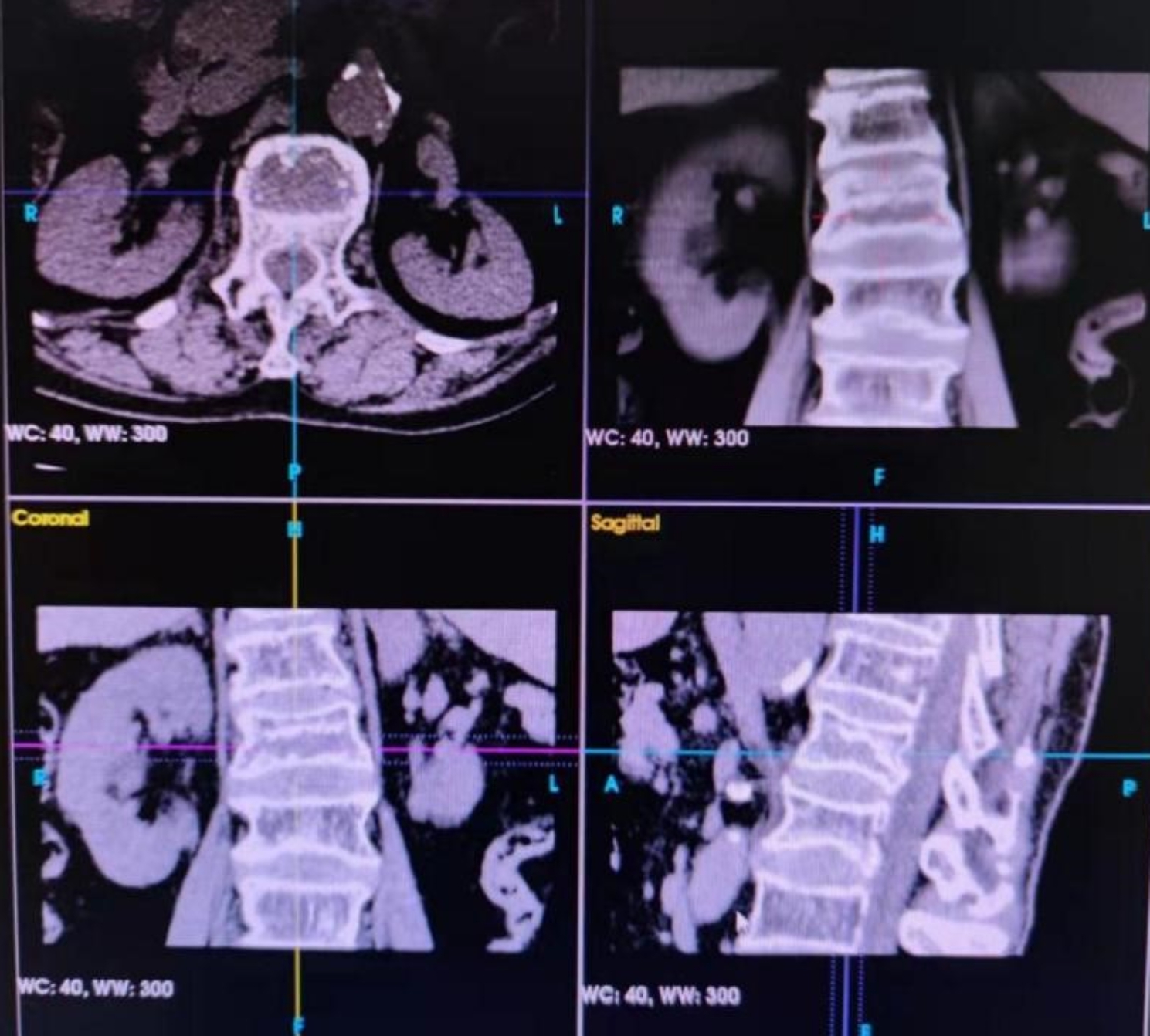
CT of a lumbar spine indicates an intact posterior wall of the lumbar 1 vertebra

**Fig. 5:**
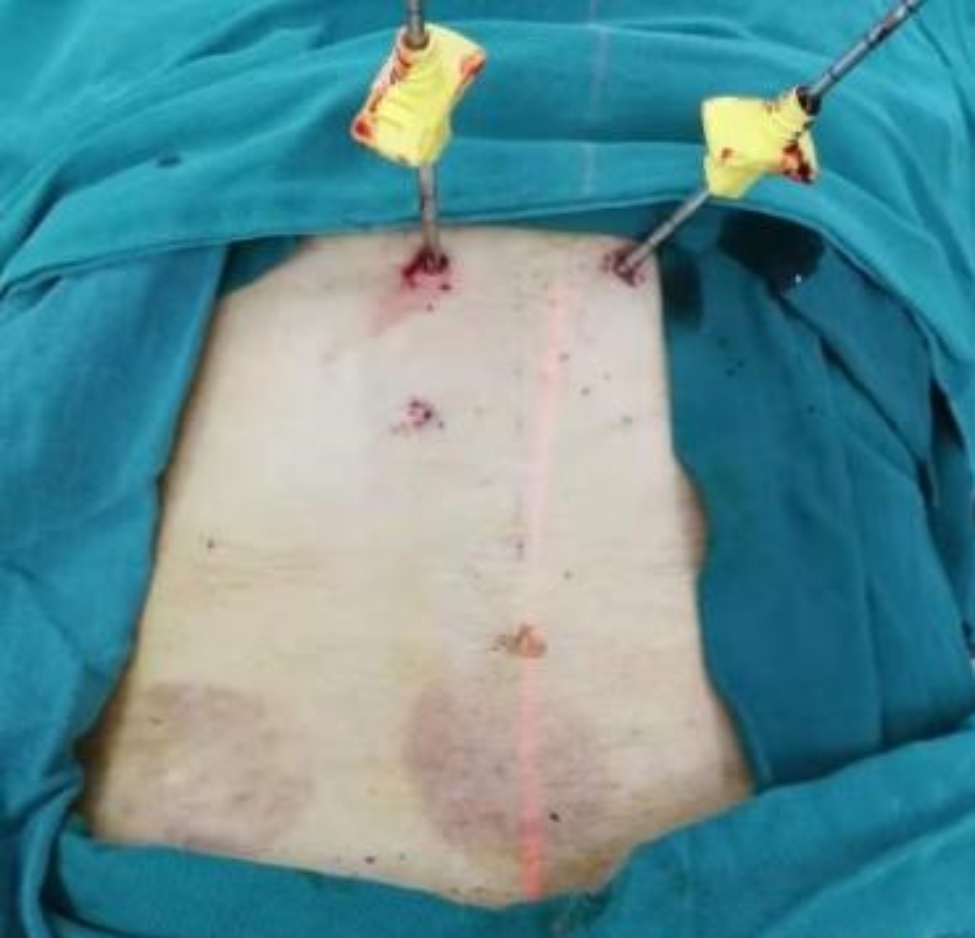
After satisfactory positioning of the lumbar 1 vertebral body, bone cement was implanted along the working channel. The patient had substantial radiating pain in the lumbosacral region before the operation and was treated with cupping at another hospital

**Fig. 6:**
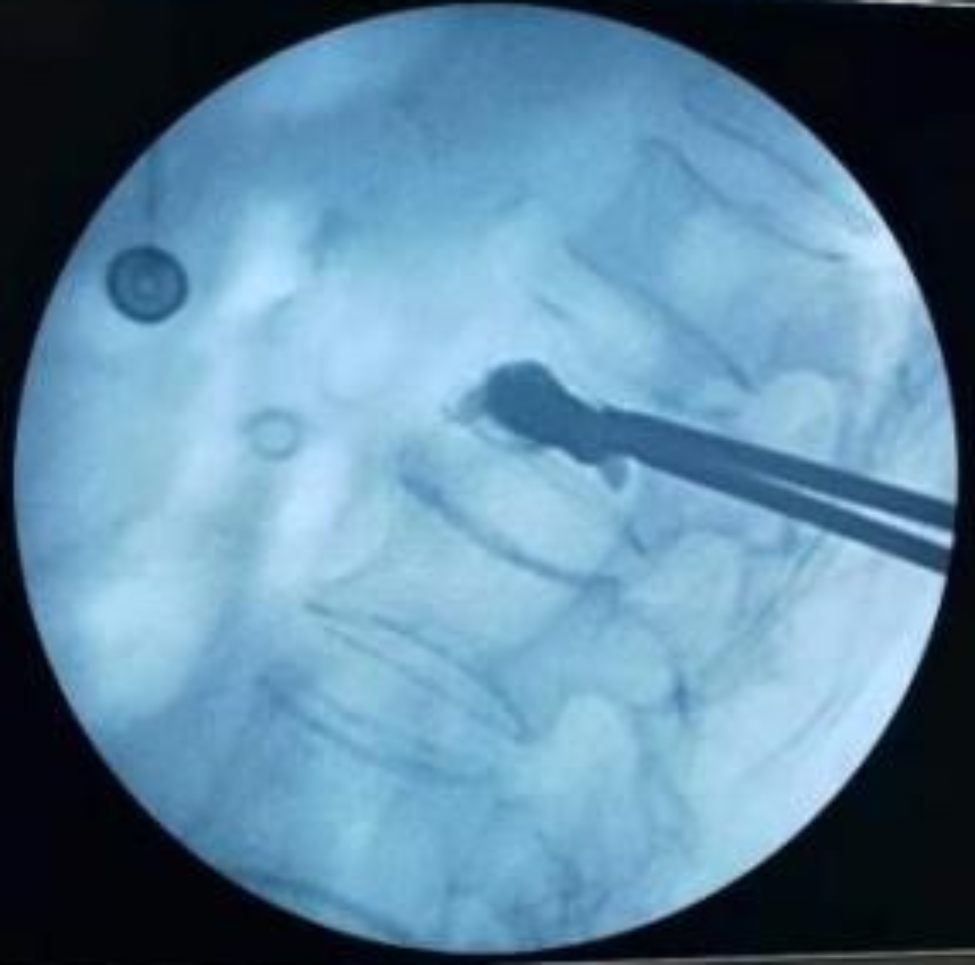
The lateral film suggests satisfactory bone cement filling

**Fig. 7:**
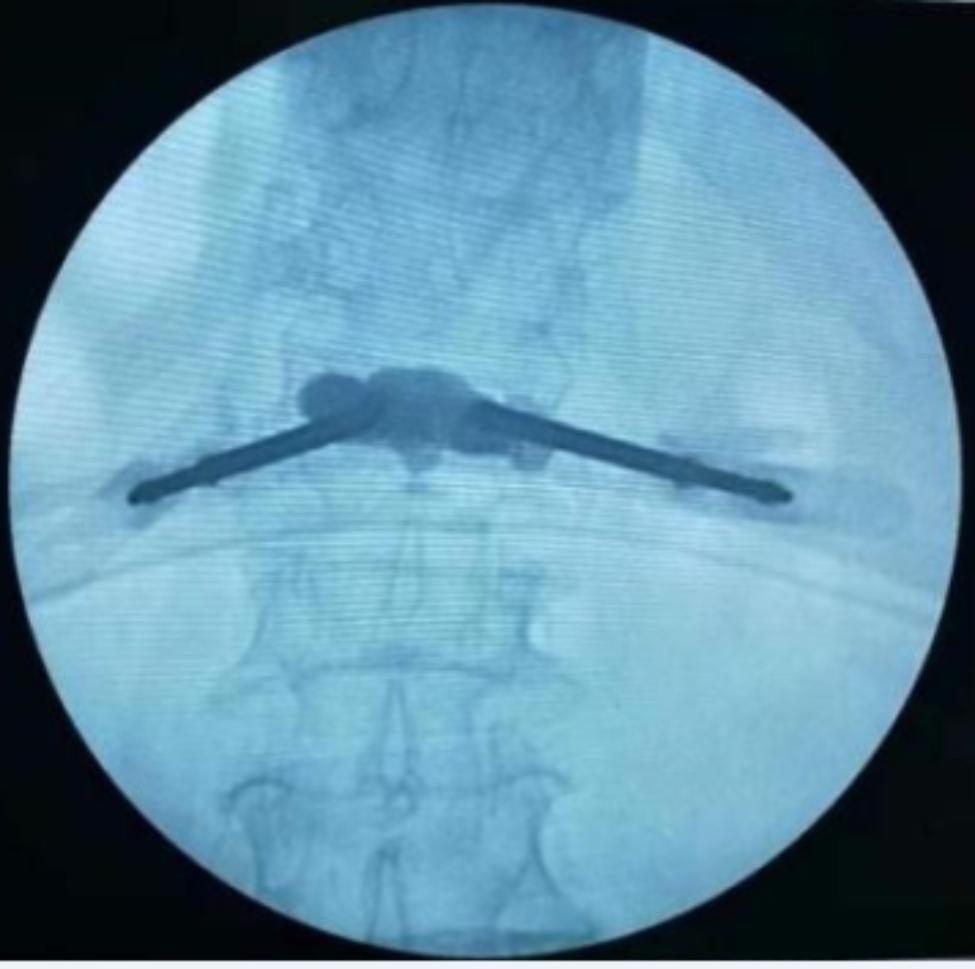
The anteroposterior film suggests satisfactory bone cement filling

**Fig. 8:**
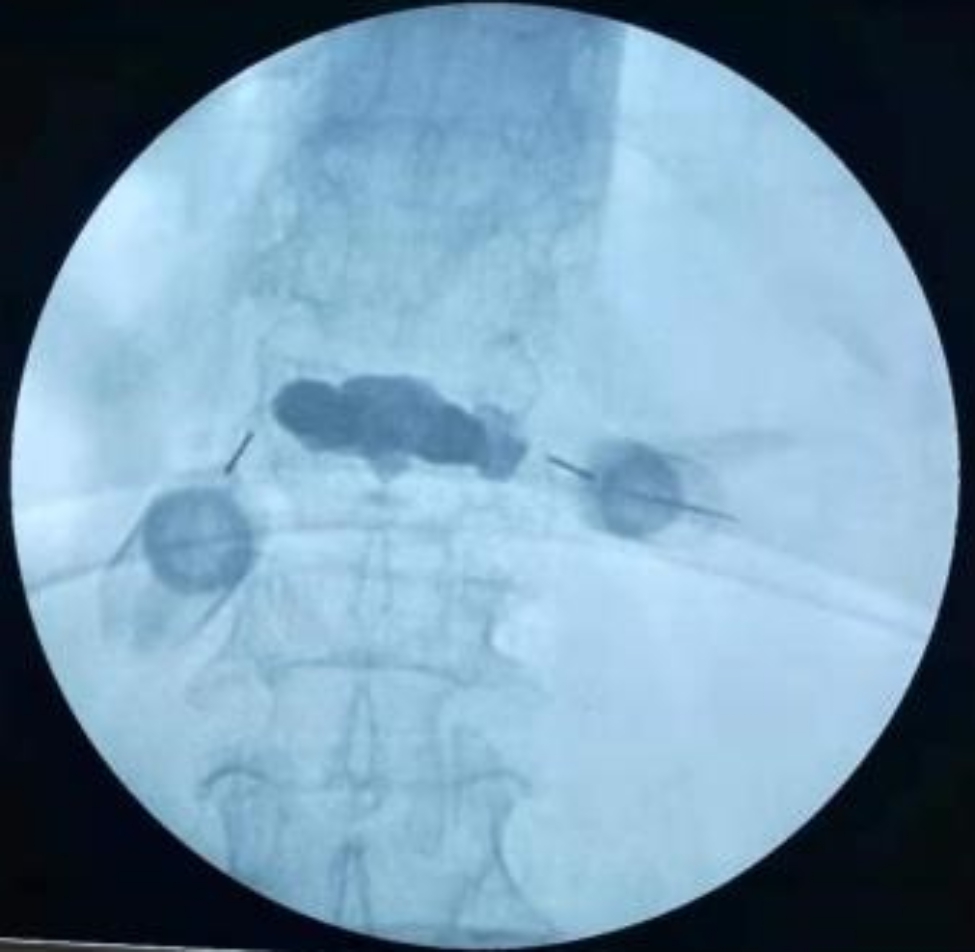
ESPB treatment was completed bilaterally using a 5 ml syringe at the root above the lumbar 1 transverse process to inject 5 ml of ropivacaine in the gap between the deep surface of the erector spinae and the transverse process

An example of a typical case is an 82-year-old female patient surnamed Yi, who was accepted to the hospital suffering from pain lower back for 48 h. A physical assessment revealed pain in the lumbar1 vertebral spine percussion and lumbosacral pressure. There was no sense of abnormality in either of her lower limbs, which had grade-5 muscle strength. An MRI assessment of the lumbar spine indicated a recent fracture of the lumbar 1. Our diagnosis before surgery was a compression fracture of the lumbar 1 vertebra and severe osteoporosis. Under the support of the G-arm radiography system, the lumbar 1 vertebral body and bilateral puncture points were located, and also placed the performing cannula with the guide needle after making a satisfactory puncture. Mixing the bone cement and filling the injured vertebra with that were accomplished after it had reached the ideal consistency. Since then, with the aid of the G-arm, we performed ESPB treatment with an injection of 5 ml of ropivacaine bilaterally at the root superior of the lumbar 1 transverse process. **(**Figs. 3, 4, 5, 6, 7 and 8**)**

The time of operation was nearly 53 min, with a 2-milliliter loss of blood during surgery. The affected people had a VAS of 8 and an ODI of 80%. Two hours after the operation the score VAS was 1, as well as the ODI index, was 24%, while she had a VAS of 1 and an ODI of 10% when she was discharged from the hospital. After returning the affected people to the ward, we measured the post-operation ambulation time. We noted that the myogenic pain in the injured vertebrae was significantly relieved.

## Discussion

In this aging society, osteoporotic vertebral compression fractures have become more commonplace [14, 15] OVCF refers to spinae breakages which take place with or without minor trauma because of declined bone strength or declined bone density, as well as bone mass caused by osteoporosis. It is primarily manifested as pain and limits chest motion, waist, and back with or without neurological signs in the lower severity, [16] and has a subtle beginning. Recently, instances of OVCF have been steadily increasing, and it has a serious effect on adults who are of higher ages and women who are in the postmenopausal period in particular. Of the 100 patients in this study, 83 were women, accounting for the vast majority [17] The pain caused by OVCF seriously impresses the life quality, activity range, and psychological well-being of affected people, while long-term resting on the bed can lead to more complications like deep vein thrombosis in the lower extremities, pressure sores, as well as pulmonary infections, in specific cases whom their lives might be very in danger [18] Related to some peer reviews, the annual rate of mortality for people who were affected by OVCF remarkably is more than such cases in the overall population [19].

The preliminary approach for the surgery for OVCF is percutaneous vertebroplasty as well as percutaneous kyphoplasty (PKP).[20] Vertebroplasty might increase the consistency as well as strength of the injured vertebrae, while the high temperature of the bone cement might inactivate the inflammatory factors in the vertebral body and decline the nociceptive sensitivity of the spinae branches. As a result, the patient’s pain might be dramatically reduced after the operation [21] However, patients often have residual postoperative muscular aches and pains distributed along the erector spinae muscle that is manifested as distal pain radiation. The results of this study indicate ESPB treatment with the assistance of vertebroplasty can significantly alleviate injured vertebral distal myogenic pain. From the point of anatomy, the erector spinae muscle is innervated by the posterior branch of the spinae nerve, which extrudes posteriorly to the intervertebral foramen and also moves distally for nearly three vertebral segments, except the sacral nerve. This explains the reasons that affected people by OVCF frequently experience a mixture of pain in the myalgic distal to the injured vertebrae. In this research, the local anesthetic the injection of ropivacaine was done deep into the surface of the erector spinae as well as the transverse process (the upper root of the transverse process) under the guidance and positioning of the G-arm radiography system. This outspread to the space of paravertebral, also obstructed the posterior branch of the spinae nerve, thereby obstructing the erector spinae as well as relieving myalgic pain [22, 23] Regarding the anatomical structure, the posterior branch of the spinae nerve that is obstructed in the area of the injured vertebra is the posterior branch of the spinae nerve which originates from underneath the area of the adjacent vertebra. However, the upper posterior branch of the spinae nerve is the one that early innervates the area of injured vertebrae, hence the obstruction is considered at the superior of the root transverse process of injured vertebrae.

Ropivacaine is the medicine for ESPB therapy, and Naropin is its financial name. Furthermore, this drug is produced by AstraZeneca AB with a concentration of 0.75% (7.5 mg/mL). Moreover, Ropivacaine indicates less heart toxicity, therefore this can be appropriate for local obstructions in affected people who are elder [24, 25] The most general concentration of this drug which is applied, is the concentrations among 0.5–1.0%, as well as the obstruction of sensory nerves, is roughly three to five hours, therefore this is swift as well as long-lasting local analgesia. The topmost dose of medicine is 200 mg (or 40 ml of 0.5% solution). We administered 5 ml of ropivacaine for every side of the injured vertebrae, also because the affected people who take part in the experiment were limited to have only two or fewer injured spinae segments, the highest total amount of ropivacaine administered considered 20 ml, which was secure and trusty as well.

The statistical differences were not before operation VAS and ODI scores between the PVP + ESPB (observation) and the PVP (control) groups. This indicates the circumstances of the patients in the two groups were very similar when they were admitted to the hospital. However, in the measurement of both scores of VAS and ODI 120 min following the operation as well as at the time of discharge from the hospital, the first group (the observation category) demonstrated noticeably fewer scores than the second group (control category), with statistically significant differences. This shows the affected people in the first group received more effective pain alleviation as well as displayed further clear recovery of the lumbar activity following the ESPB therapy. Additionally, ESPB showed a remarkable impact on the amelioration of the pain of erector spinae. Based on the test conclusions, some statistical differences were between both groups in early ambulation time after the operation as well as the time of defecation. It demonstrates the pain symptoms of affected people in the observation category developed earlier and sooner with ESPB-assisted therapy, also affected people were able to walk sooner and experience bowel mobility more rapidly. Based on the outcomes, there were no statistical differences between the two categories from the point of the operation term, bone cement repletion volume during operation, loss of blood during the surgery, as well as operating expenses. This confirms that the ESPB therapy was not more invasive, nor did it rise the operation term, loss of blood during surgery, or operation costs. Besides, there did not have statistical differences in the quantity of bone cement filling during the surgery used between both categories, also the occurrence of complications in each group after the operation. Based on these findings, ESPB did not rise the operation term, the risk of surgery, or the financial burden on affected people in comparison to PVP treatment alone. By diffusing the local anesthetic medicine ropivacaine into the paravertebral space, it obstructs the posterior branch of the spinae nerve, and further consoles the rest of the myalgic pain, as well as pain, thus promoting amelioration swiftly.

In 2016, Ferero et al. [22] first reported on ESPB therapy, which was used in analgesic therapy for the pain of thoracic neuropathy. ESPB is a novel method of trunk nerve block that has the advantages of low risk, minor trauma, also remarkable influence. It has already been applied for perioperative analgesia for the operation of the thoracic, breast, and abdominal. However, there are only a few reports on the usage of ESPB for spinae operation, and there have been limited clinical trials. However, considering its benefits, many scholars believe that it will soon be vastly applied to OVCF affected as well as other parts of spinae operation [26, 27] In terms of the therapy of chest, waist, and back chronic pain, there are plenty of affected people with chronic pain in these sectors, yet imaging assessment frequently does not show any remarkable abnormalities in the chest, waist, and back. Tension in the erector spinae muscle is found via physical assessment with pain manifesting under pressure. Recently, researchers have indicated the posterior branch of the spinae nerve has a key role in chronic pain of spinae origin [28] ESPB immediately obstructs the posterior branch of the spinae nerve, as a result of that mitigating tension in the erector spinae muscle as well as mitigating pain in the chest, waist, and back. Regarding routine perioperative analgesia in the operation of spinae, lumbar spondylolisthesis, lumbar disc herniation, lumbar spinae stenosis, and thoracolumbar fractures are common diseases and frequently need further operation therapy. Spinae operation is invasive and prolonged with further pronounced pain after surgery than general operation. Deng Lin et al. [29] Performed the ultrasound-guided vertical spinae muscle block in the T7 plane during posterior lumbar surgery, as an adjunct to general anesthesia. They suggested that ESPB provides after-operation analgesia, reduces the dose of analgesic pump medicines as well as more analgesics, and also improves patients’ consent due to postoperative analgesic influence.

A limitation of this study is the bias in results measurement. Because the pain threshold of each patient is not the same and also the perception of pain is different, the measurement results of individual patients may be biased.

## Conclusion

As a novel nerve-blocking method, ESPB has a superficial point of action, and it is a way of vital organs and blood vessels, contributing to very slight complications and risks such as pneumothorax, hematoma, and nerve damage. ESPB indicates the additional benefits of safety, comfortable and easy surgery, low toxicity, also few side effects. Moreover, it has a significant adjunctive analgesic influence on affected individuals who have undergone percutaneous vertebroplasty likewise the probability of exploiting a positive therapeutic effect in other parts of spinae operation.

## Data Availability

All data will be available upon reasonable request to the corresponding author.
